# Blastic Plasmacytoid Dendritic Cell Neoplasm Developed in Chronic Myeloid Leukemia in Molecular Remission During a Four-Year Treatment-Free Interval After Six Years of Dasatinib Treatment

**DOI:** 10.7759/cureus.61944

**Published:** 2024-06-08

**Authors:** Hiroshi Suzuki, Masataka Takeshita, Risen Hirai, Akira Tanimura, Akiyoshi Miwa

**Affiliations:** 1 Department of Hematology, Tokyo Kita Medical Center, Tokyo, JPN

**Keywords:** dasatinib, chronic myeloid leukemia, cd4+ cd56+ natural killer hematolodermic neoplasms, blastic natural killer cell lymphoma, agranular cd4+ natural killer cell leukemia, blastic plasmacytoid dendritic cell neoplasm

## Abstract

Blastic plasmacytoid dendritic cell neoplasm (BPDCN) is a rare hematological malignancy affecting multiple sites, most commonly the skin. About 10-20% of BPDCN cases are accompanied by hematological neoplasms. A 71-year-old male was diagnosed with chronic myeloid leukemia in the chronic phase (CML-CP) 11 years prior (at 60 years of age), and dasatinib treatment was initiated. A major molecular response (MMR) was achieved 18 months after diagnosis, and the molecular response (MR)^4.0^ lasted beyond 36 months. Due to pancytopenia, dasatinib was discontinued at 74 months, but the CML-CP remained undetectable. One hundred and twenty-two months after the diagnosis, the patient presented with cutaneous lesions on the forehead and abdomen. Immunological and histological analyses of the skin biopsy showed infiltration of atypical cells from the deep epidermis to the entire dermis, expressing clusters of differentiation (CD) 4, CD56, and CD123 without any other markers. The same cells were observed in bone marrow samples. BPDCN was diagnosed, followed by chemotherapy and possibly autologous or allogeneic hematopoietic stem cell transplantation (HSCT). To the best of our knowledge, this is the first case report of the development of BPDCN in a patient with CML in molecular remission. Further studies are required to clarify the pathogenesis of BPDCN in patients with hematological malignancies in remission.

## Introduction

Blastic plasmacytoid dendritic cell neoplasm (BPDCN) is a rare and aggressive hematological malignancy thought to be derived from the precursors of plasmacytoid dendritic cells [1]. The causes of BPDCN are unknown and have a frequency of 0.04 cases per 100,000 populations [2]. Males are predominantly affected, with a ratio of approximately 3:1 and a median age at diagnosis of 69 [3]. BPDCN is characterized by a high frequency of cutaneous lesions; other sites include bone marrow, lymph nodes, spleen, and peripheral blood.

Some studies suggest associations with BPDCN and other myeloid malignancies [3,4], including very few cases of chronic myeloid leukemia (CML). To the best of our knowledge, this is the first case report of BPDCN that developed in cutaneous lesions, lymph nodes, and bone marrow in a patient with CML who had been in molecular remission for four years since the cessation of dasatinib.

## Case presentation

Eleven years prior, leukocytosis was detected in a 60-year-old male during an annual health checkup. He reported no fever, weight loss, night sweats, skin lesions, or other symptoms. He had no medical history of radiation exposure or use of organic solvents. On physical examination, vital signs were normal, and he had no remarkable findings, including hepatosplenomegaly or lymphadenopathies. Laboratory studies showed a leukocyte count of 106,070/µL, with 54% neutrophils, 6% lymphocytes, 1% eosinophils, 3% basophils, 9% metamyelocytes, 26% myelocytes, and 1% myeloblasts. The thrombocyte count was 601,000/µL in the peripheral blood. The kidney and liver function tests were normal based on biochemical analyses. Other laboratory test results are presented in Table 1. Computed tomography (CT) scans revealed a 10-cm spleen with a vertical span without hepatomegaly or lymphadenopathy. A bone marrow biopsy revealed hypercellular marrow with granulocyte hyperplasia at various maturation stages, without hiatus leukemicus. The myeloblast count was 0.6% of the nucleated marrow cells. Cytogenetics analysis showed karyotype 46, XY, t(9;22)(q34;q12.2). Fluorescence in situ hybridization (FISH) revealed 97% positivity for BCR-ABL fusion signals. A bone marrow biopsy revealed no prominent marrow fibrosis.

**Table 1:** Laboratory data of the first examination. AST: asparate aminotransferase, ALT: alanine aminotransferase, LDH: lactate dehydrogenase, ALP: alkaline phosphatase, ɤ-GTP: gamma-glutamyl transpeptidase, CRP: C-reactive protein, BNP: brain natriuretic peptide, PT: prothrombin time, aPTT: activated partial thromboplastin time.

Lab	Results	Unit	Reference range
White blood cells	106,070	/µL	3,500–8,500
Myeloblast	1	%	
Myelocyte	26	%	
Metamyelocyte	9	%	
Neutrophils	54	%	27–91
Eosinophils	1	%	0–7
Basophils	3	%	0–2
Monocytes	1	%	1–8
Lymphocytes	6	%	18–50
Hemoglobin	12.6	g/dL	13.5–17.0
Platelet	628,000	/µL	150,000–350,000
Total protein	6.9	g/dL	6.6–8.1
Albumin	4.1	g/dL	3.5–5.0
Total bilirubin	0.6	mg/dL	0.4–1.5
AST	29	U/L	13–33
ALT	28	U/L	8–42
LDH	896	U/L	119–229
ALP	236	U/L	115–359
ɤ-GTP	59	U/L	10–47
Uric acid	7.1	mg/dL	3.6–7.0
Urea nitrogen	12.7	mg/dL	8.0–22.0
Creatinine	0.53	mg/dL	0.60-1.10
Na	135	mEq/L	138-146
Cl	100	mEq/L	99-109
K	4.1	mEq/L	3.6-4.9
Ca	9.4	mg/dL	8.5-10.5
P	3.0	mg/dL	2.5-4.7
Glucose	117	mg/dL	69-104
CRP	0.18	mg/dL	0-0.3
Vitamin B12	3,444	pg/mL	233-914
IgG	1132	mg/dL	861-1747
IgA	235	mg/dL	93-393
IgM	72	mg/dL	33-183
Ferritin	482.2	ng/mL	21-274
BNP	20.4	pg/mL	up to 18.4
PT	12.3	Second	10.4-14.1
aPTT	27	Second	24.0-38.0
Fibrinogen	364.1	mg/dL	200.0-400.0

**Figure 1:**
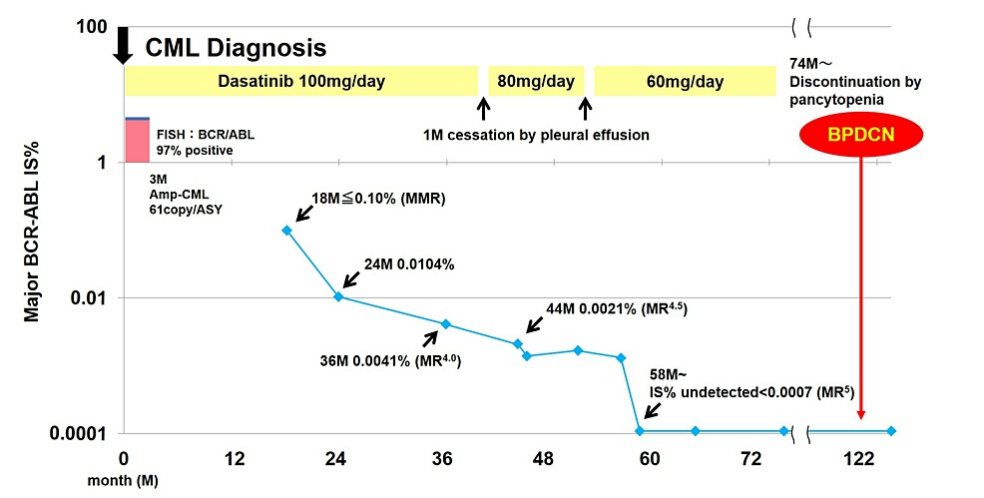
Clinical course of BCR-ABL IS% from diagnosis of CML to diagnosis of BPDCN. IS: international scale, CML: chronic myeloid leukemia, BPDCN: blastic plasmacytoid dendritic cell neoplasm, FISH: fluorescence in situ hybridization, MMR: major molecular response, MR: molecular response.

CML in the chronic phase (CML-CP) was diagnosed, and the Sokal score was calculated to be 0.21, suggesting low risk. Dasatinib (100 mg/day) was administered, which resulted in a major molecular response (MMR) and molecular response (MR)^4.0^ at 18 and 36 months, respectively. At 42 months, dasatinib was discontinued for one month due to pleural effusion and restarted at 80 mg/day after improvement of the pleural effusion. After the second one-month cessation due to pleural effusion at 55 months, 60 mg/day of dasatinib was reinitiated, and MR^5.0^ was achieved at 58 months. Due to pancytopenia, dasatinib was discontinued at 74 months; however, the International Scale (IS) score for CML-CP remained at undetected levels (Figure 1).

**Figure 2:**
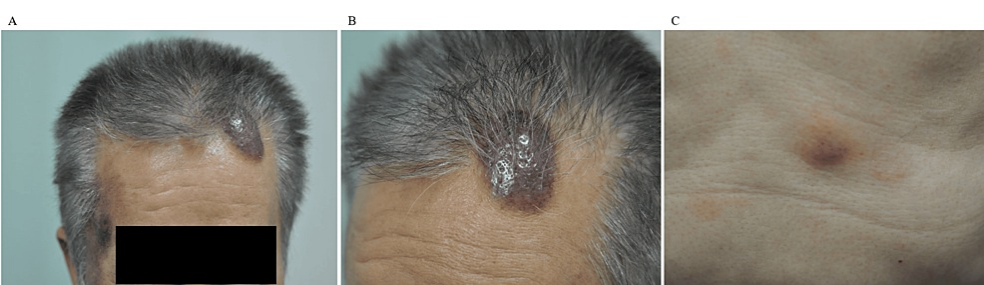
Cutaneous lesions on forehead (A, B) and abdomen (C).

**Figure 3:**
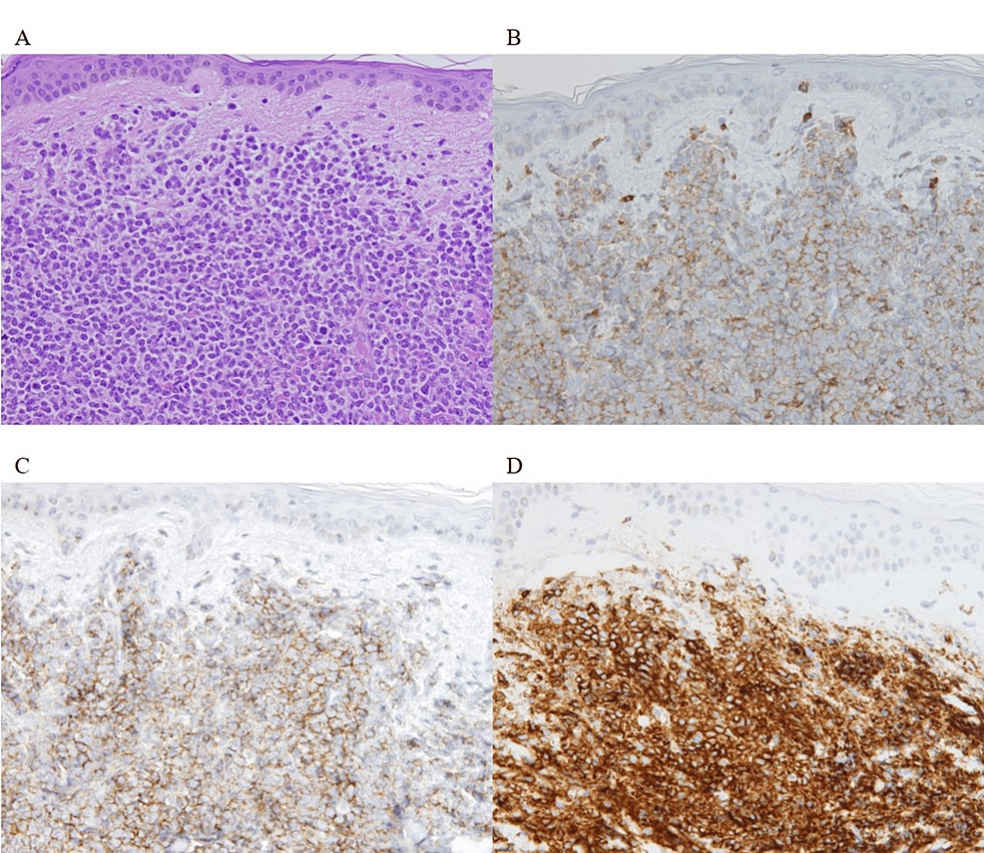
(A) Skin biopsy of abdomen showing malignant cells from deep epidermis to entire dermis (hematoxylin and eosin staining, ×400). The malignant cells show positive CD4 (B), CD56 (C), and CD123 (D). CD: cluster of differentiation.

**Figure 4:**
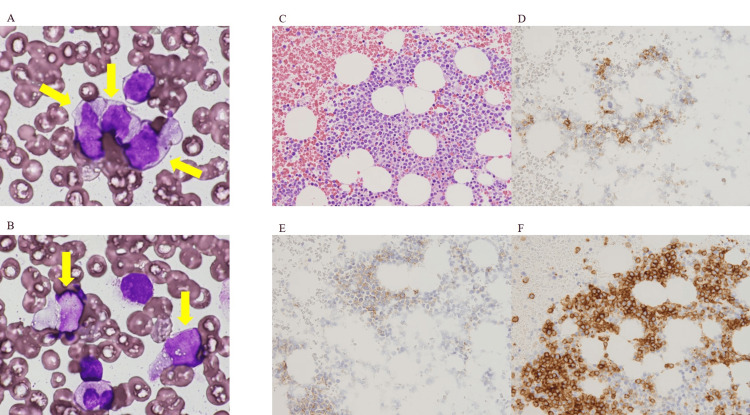
(A,B) Bone marrow aspiration and biopsy showing 13.0% malignant cells (arrow, May-Giemsa staining, ×1,000), with similar cells (C) (hematoxylin and eosin staining, ×400) and the same immunophenotype as cutaneous lesion [CD4 (D, weak positivity), CD56 (E, weak positivity), and CD123 (F)]. CD: cluster of differentiation.

At 122 months after diagnosis (48 months after cessation of dasatinib), cutaneous lesions were detected on the forehead and abdomen (Figure 2). Although CML remained in remission despite dasatinib cessation, skin disease development was observed. Among the various possible causes, CML-related skin changes were considered. Therefore, a skin biopsy and bone marrow aspiration were performed. Immunohistochemistry revealed that malignant cells from the deep epidermis to the entire dermis displayed positive immunoreactivity for a cluster of differentiation (CD) 4, CD56, and CD123 and negative immunoreactivity for myeloperoxidase (MPO), CD11c, CD3, and CD79a (Figure 3). Bone marrow aspiration and biopsy revealed 13.0% of malignant cells with the same immunophenotype as the cutaneous lesion (Figure 4). Cytogenetic test results were normal, and IS was below the detectable limit. CT scan revealed left inguinal lymphadenopathy without hepatosplenomegaly. Based on these findings, the diagnosis of BPDCN was made, and the major sites of BPDCN infiltration were the skin and bone marrow. We could not prove the histological involvement of BPDCN in the lymph node because a lymph node biopsy was not performed.

**Figure 5:**
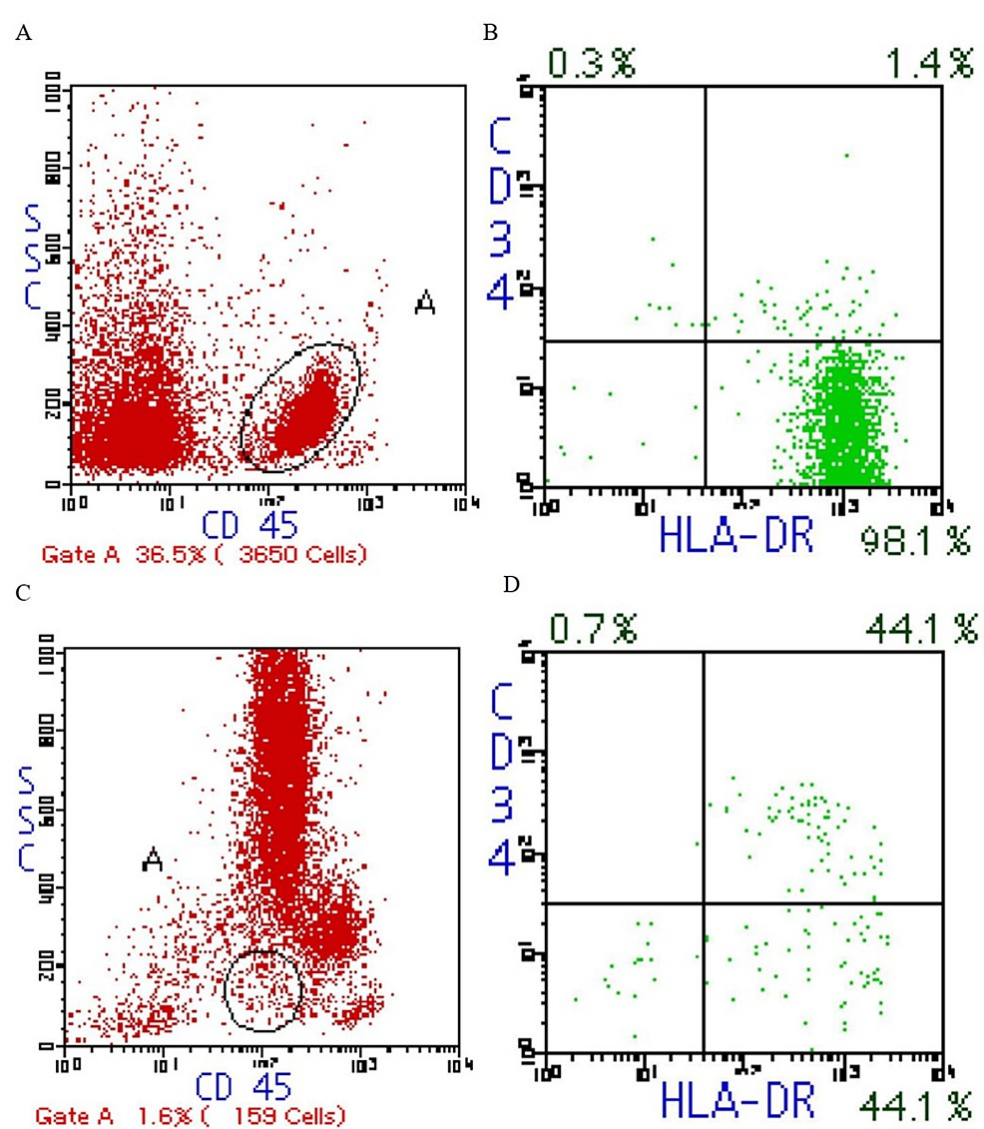
Flow cytometry showing HLA-DR (+) and CD34 (−) malignant cells reduced from 98.1% at diagnosis (A,B) to 44.1% after the first cycle of MA (C,D). HLA: human leukocyte antigen, CD: cluster of differentiation, MA: methotrexate and cytarabine.

**Figure 6:**
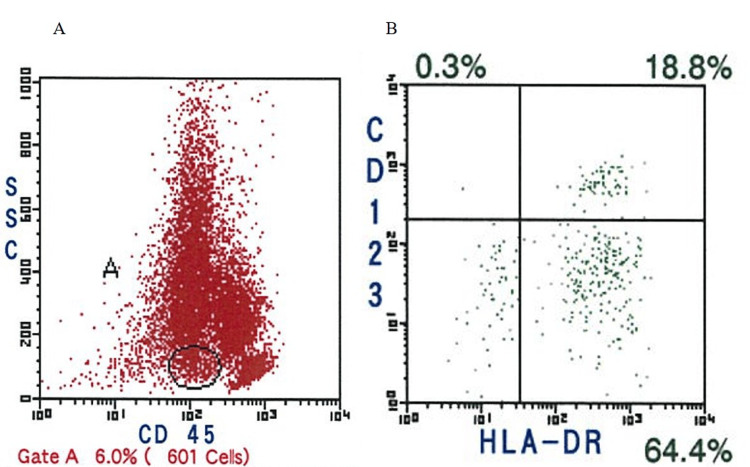
Flow cytometry showing collected cells on PBSCH (A) and CD123 (+) and HLA-DR (+) malignant cells account for 1.1% of them (B). PBSCH: peripheral blood stem cell harvest, CD: cluster of differentiation, HLA: human leukocyte antigen.

The rarity of the association between CML in remission and BPDCN was confirmed using a MEDLINE search and other online surveys. Additionally, no standardized regimens have been reported for treating these combinations. However, we elaborated on the treatment for this patient. Chemotherapy was planned as the first-line treatment. Through an online survey, we selected hyper-CVAD and MA (cyclophosphamide, vincristine, doxorubicin, dexamethasone [CVAD] and methotrexate and cytarabine [MA]) regimens based on the report by Tsagarakis and Pagano [5,6]. Following chemotherapy, we planned autologous or allogeneic hematopoietic stem cell transplantation (HSCT). The hyper-CVAD and MA regimens were initiated. After the first cycle of hyper-CVAD, skin lesions on the forehead and abdomen showed rapid shrinkage with residual skin pigmentation, and the left inguinal lymph node enlargement disappeared. Bone marrow aspiration performed after the first cycle of MA showed 12.0% malignant cells, but human leukocyte antigen (HLA)-DR (+) and CD34 (−) cells, which were the immunohistochemical finding patterns on malignant cells, reduced from 98.1% (at diagnosis) to 44.1% (Figure 5). Peripheral blood stem cell harvest (PBSCH) was planned after the second hyper-CVAD cycle. CD34 (+) cells collected on PBSCH accounted for 14.41 × 106 per kg, but CD123 (+) and HLA-DR (+) malignant cells accounted for 1.1% (Figure 6). Therefore, we did not use them and planned allogeneic HSCT during the subsequent hyper-CVAD/MA regimen cycles. Before the second cycle of MA, bone marrow aspiration showed complete remission (CR), with minimal residual malignant cells in 1.0% of marrow nucleated cells.

Bone marrow aspiration performed before the third cycle of MA showed steady CR, but a slight increase in the number of malignant cells (3.4%) was observed. We recommended allogeneic HSCT; however, the patient wished to avoid it because of the adverse events. Therefore, we decided not to perform allogeneic HSCT but to perform another cycle of hyper-CVAD/MA through a shared decision-making process. After the fourth cycle of MA, chemotherapy was terminated, and periodic follow-up was initiated.

## Discussion

BPDCN is an aggressive hematologic malignancy of plasmacytoid dendritic cells, as classified by the World Health Organization (WHO) [1]. It was formerly termed agranular CD4 (+) natural killer (NK) cell leukemia [7] and later blastic NK cell lymphoma [8], and finally as CD4 (+) CD56 (+) NK hematolodermic neoplasms [9]. The WHO named it BPDCN, classified it as acute myeloid leukemia (AML) and related precursor neoplasms in 2008, and established BPDCN as its own distinct entity in 2016 [1].

The diagnosis of BPDCN is often based on the immunophenotype. BPDCN cells can be identified based on the following phenotypes: CD45 (+, low), CD4 (+), CD56 (+), CD116 (+, low), CD123 (+, high) (interleukin-3α receptor), HLA-DR (+), CD45RA (+), CD45R0 (−), BDCA-2 (blood dendritic cell antigen-2 or CD303) (+), BDCA-4 (CD304) (+), and ILT-3 (immunoglobulin-like transcript-3) (+) [10]. Julia et al. suggested that a diagnosis of BPDCN is made by positive immunoreactivity for four of five markers (CD4, CD56, CD123, CD303, and TCL1 [T-cell leukemia/lymphoma 1]) and revealed that approximately 50% of patients showed simultaneous expression of these five markers [11]. Garnache-Ottou et al. suggested a scoring system for the diagnosis of BPDCN that requires the investigation of BDCA-2, CD123, and BDCA-4 expression and a limited number of lineage-specific markers (CD4, CD56, CD11c, MPO, cytoplasmic CD79a, and cytoplasmic CD3) [12]. Therefore, diagnosing BPDCN requires positive immunoreactivity for CD4, CD56, and CD123 and negative immunoreactivity for lineage-specific markers. Our case fulfilled this, and a diagnosis of BPDCN was made by skin and bone marrow biopsies.

Traditionally, multi-agent chemotherapy regimens designed for acute leukemia, lymphoma, and myeloma have been used for BPDCN, and induction regimens for acute leukemia have been reported to achieve slightly better outcomes [3,13]. The hyper-CVAD/MA regimen is one of the strategies generally used for several lymphoid neoplasms, resulting in a somewhat high or high response rate; however, responses are generally transient [5,6]. HSCT is a curable treatment for BPDCN, and both autologous and allogeneic HSCT in initial CR appear to be reasonable treatment options [14,15]. Tagraxofusp, a CD123-directed cytotoxin consisting of recombinant human interleukin-3 fused to a truncated diphtheria toxin, has been an integral agent for patients with both newly diagnosed and relapsed/refractory BPDCN since its Food and Drug Administration (FDA) approval in December 2018. In Japan, a phase I/II clinical trial of Tagraxofusp for BPDCN (Japanese Registry of Clinical Trials: jRCT2031220023) was ongoing, but our case was not eligible because of the small amount of residual tumor.

As shown in this context, various reports have described BPDCN development in patients with prior blood malignancies. In contrast, some reports have shown the development of hematological malignancies after the initial diagnosis of BPDCN. Some studies revealed a relationship between BPDCN and other hematologic malignancies and showed 8.7% to 20% of BPDCN patients had medical histories of them (Table 2) [3,4,6,16]. In these studies, only Julia et al. reported six cases of BPDCN with a history of CML in all 90 cases [16]. Although BPDCN is a lethal disease, some cases have been reported after diagnosis (Table 3) [17-19]. Ramachandran et al. investigated the risk of secondary primary malignancies (SPMs) in patients with initial BPDCN using the National Cancer Institute’s Surveillance, Epidemiology, and End Results (SEER) database [19]. In 932 patients with BPDCN, SPMs occurred in 43 patients and hematologic malignancies in 12 patients; however, they did not include CML patients.

**Table 2:** BPDCN and medical histories of other hematologic malignancies. BPDCN: blastic plasmacytoid dendritic cell neoplasm, MDS: myelodysplastic syndrome, FL: follicular lymphoma, NHL: non-Hodgkin lymphoma, CML: chronic myeloid leukemia, MM: multiple myeloma, ET: essential thrombocythemia.

References	Country	Number of BPDCN	Medical histories and number of them (%)
Feuillard et al. [3]	France	23	MDS 2 (8.7)
Reichard et al. [4]	United States	10	MDS 1 (10.0), FL 1 (10.0)
Pagano et al. [6]	Italy	43	MDS 4 (9.3), NHL 2 (4.7)
Julia et al. [16]	France	90	CML 6 (6.7), MDS 3 (3.3), MM 1 (1.1), ET 1 (1.1)

**Table 3:** Other hematologic malignancies that occurred after the diagnosis of BPDCN. BPDCN: blastic plasmacytoid dendritic cell neoplasm, AML: acute myeloid leukemia, NHL: non-Hodgkin lymphoma, CLL: chronic lymphocytic leukemia, AMMoL: acute myelomonocytic leukemia.

References	Country	Number of BPDCN	Hematologic malignancies and number of them
Khoury et al. [17]	United States	7	AML 2
Herling et al. [18]	United States	12	AML 3
Ramachandran et al. [19]	United States	932	NHL 1, CLL 1, AML 7, AMMoL 1, other leukemia 1

From this perspective, multistep carcinogenesis may be related to the development of BPDCN in non-BPDCN hematological malignancies. Genetic instability or other background factors may also be linked to this condition. Some reports on AML genesis and non-CD33 marrow stromal elements have shown chromosomal abnormalities, indicating the presence of genetic instability or predisposition as a background for leukemogenesis [20]. Considering the various possible pathways, further analyses are required. In this case, the contribution of dasatinib to the modification of clinical pictures may be a new theme.

## Conclusions

In conclusion, we emphasize the rarity of our case: BPDCN developed after four years of no treatment in a patient with CML-CP treated for six years with dasatinib. The mechanisms of action of BPDCN in other hematological malignancies have not been fully investigated. Further studies, including those on genetic instability, are required to clarify the pathogenesis of BPDCN in patients with hematological malignancies in remission.
